# IL1B-centered immune dysregulation involving IL7R, CCR7, ITGB2 and IRF1 across insomnia and inflammatory bowel disease

**DOI:** 10.3389/fimmu.2026.1829141

**Published:** 2026-09-01

**Authors:** Han Guo, Xudong Xu, Huanying Shi, Meng Cui, Min Zhang, Hongdan Wang, Yunxuan Zhang, Haifeng Zhou, Xu Sun, Xiaoyan Liu

**Affiliations:** 1 Department of Pharmacy, Huadong Hospital, Fudan University, Shanghai, China; 2 Department of Digestive Diseases, Huashan Hospital, Fudan University, Shanghai, China; 3 Department of Pharmacy, Huashan Hospital, Fudan University, Shanghai, China; 4 Medical Genetic Institute of Henan Province, Henan Provincial People’s Hospital, Zhengzhou University People’s Hospital, Zhengzhou, China

**Keywords:** bioinformatics analysis, IL1B, immune cell infiltration, inflammatory bowel disease, insomnia

## Abstract

**Background:**

Insomnia is a prevalent sleep disorder that strongly affects one’s quality of life and physical well-being. Inflammatory bowel disease (IBD) is a chronic inflammatory condition of the intestines, and a majority of IBD patients suffer from comorbid insomnia. However, the shared molecular features linking insomnia and IBD remain poorly characterized.

**Methods:**

Common differentially expressed genes (DEGs) were identified in datasets of insomnia (GSE208668) and IBD (GSE179285) using the Limma package. Functional enrichment was performed by Gene Ontology (GO) and Kyoto Encyclopedia of Genes and Genomes (KEGG) pathway analyses. Protein–protein interaction (PPI) network construction and hub gene identification was subsequently performed. Furthermore, we validated the reliability of the hub genes using qRT–PCR and Enzyme-linked immunosorbent assay (ELISA). In addition, we constructed a TF–miRNA regulatory network of hub genes and assessed the abundance of immune cell infiltration in insomnia and IBD using CIBERSORT, EPIC, and xCell algorithms. Finally, we utilized the DsigDB to predict potential therapeutic candidates.

**Results:**

The analysis revealed 75 upregulated and 32 downregulated common DEGs. Functional enrichment analysis revealed the inflammatory response and immune activation as pivotal drivers underlying the pathogenesis of both insomnia and IBD. Five hub DEGs, namely, IL1B, IL7R, CCR7, ITGB2, and IRF1, were subsequently screened and validated. The TF–miRNA–mRNA regulatory network consisted of 5 TFs, 14 miRNA nodes and 5 core mRNA nodes. Immune cell infiltration analysis revealed several patterns shared between insomnia and IBD. Additionally, 10 potential therapeutic drugs for insomnia and IBD were proposed.

**Conclusion:**

Integrative coexpression network analysis reveals convergent dysregulation of an IL1B-centered immune module (comprising IL7R, CCR7, ITGB2, and IRF1) across insomnia and IBD, a shared immune disturbance and candidate targets for simultaneous intervention upon further mechanistic validation.

## Introduction

Insomnia is a highly prevalent sleep disorder characterized by difficulty falling or remaining asleep and corresponding daytime dysfunction (1). Up to one-third of the adult population worldwide experiences transient insomnia symptoms—such as early awakening and nonrestorative sleep—at any given time (1, 2). Moreover, up to 50% of older adults report such sleep disturbances, with 10%–25% meeting the diagnostic criteria for insomnia disorder (3).

Inflammatory bowel disease (IBD) is a chronic, relapsing gastrointestinal disorder that develops in genetically predisposed individuals who are exposed to environmental risk factors (4, 5). It can be divided into two main types, namely, Crohn’s disease (CD) and ulcerative colitis (UC), and affects an increasing number of individuals worldwide (6).

Sleep disturbances are highly prevalent in patients with IBD, affecting approximately 75% of those with active disease, with insomnia being the primary manifestation (7). Research has indicated that poor sleep not only predicts an increased risk of symptom flares, surgery, and hospitalization but is also independently associated with a significant decline in quality of life among IBD patients. Notably, patients widely believe that sleep issues directly trigger or worsen their gastrointestinal symptoms (7). Studies have shown that approximately 70% of IBD patients experience sleep disorders, with insomnia being the most common type, affecting 58–62% of this population—far exceeding the prevalence in the general population (6–20%). This high prevalence persists both during active disease and in remission, indicating that the underlying mechanism is not merely a consequence of symptomatic disruption but is also closely linked to sustained immune system activation driven by subclinical inflammation (8). Another cross-sectional study revealed that 50% of individuals who suffer from IBD are likely to meet the criteria for insomnia disorder, with even greater rates in those who experience active IBD (9). Collectively, this evidence highlights that insomnia is extremely common in patients with IBD and closely linked to a worsened overall disease course. Currently, research on IBD and insomnia is primarily epidemiological in nature. Investigations into the underlying mechanisms, especially genetic factors, are relatively sparse.

Through transcriptomics and bioinformatics, the identification of common transcriptional features in IBD and insomnia enables a more precise forecast of molecular pathogenesis and speeds up precision medicine. In this study, the common differentially expressed genes (DEGs) shared between the two diseases were identified using data from public databases. Functional enrichment analysis was performed on the common DEGs, followed by the identification of hub genes. We experimentally validated IL1B, IL7R, CCR7, ITGB2 and IRF1 as key hub genes in animal models; performed functional annotation and multilayer gene-regulatory network analysis; characterized the associated immune responses; and predicted potential therapeutic drugs. This study aims to elucidate the shared molecular underpinnings of insomnia and IBD through integrative coexpression network analysis and identify candidate targets for concurrent therapeutic modulation.

## Methods

### Raw data download

The National Center for Biotechnology Information (NCBI) Gene Expression Omnibus (GEO) datasets (http://www.ncbi.nlm.nih.gov/geo/) were utilized to obtain second-generation high-throughput sequencing data of *Homo sapiens* and microarray data for various illnesses. We searched for gene expression datasets related to insomnia and IBD by using these terms as keywords and downloaded microarray datasets from the NCBI GEO public database. The GSE208668 Series Matrix File (the peripheral blood samples of 17 insomnia patients and 25 controls) was downloaded from the NCBI GEO public database, and the licence for the annotation platform was GPL10904 (3). The GSE179285 Series Matrix File was downloaded, with annotation under GPL6480 (10). The original dataset comprised biopsies from multiple anatomical sites and disease states: sigmoid colon, ascending/descending colon, and terminal ileum from healthy controls; uninflamed and inflamed regions of the ascending/descending colon and terminal ileum from CD patients; and uninflamed and inflamed sigmoid colon from UC patients. To eliminate confounding biases from anatomical heterogeneity and uninflamed tissue background, we strictly screened samples and included only healthy intestinal specimens and inflamed lesions from typical IBD subtypes for subsequent differential analysis. Specifically, the final cohort comprised 31 biopsies from 12 unique healthy individuals (12 uninflamed ascending/descending colon, 11 uninflamed sigmoid colon, and 8 uninflamed terminal ileum, with 11 individuals contributing multiple intestinal sites) and 70 biopsies from 65 unique IBD patients (33 inflamed terminal ileum and 14 inflamed ascending/descending colon from 42 unique CD patients; 1 inflamed ascending/descending colon and 22 inflamed sigmoid colon from 23 unique UC patients). Among the IBD cohort, three CD patients contributed multiple biopsies: two patients provided both colonic and ileal biopsies, and one patient contributed four replicate colonic biopsies (**Supplementary File 1**, all samples are annotated with corresponding patient identifiers).

### Identification and functional enrichment analysis of DEGs

Within each dataset, raw expression data were preprocessed as follows: probe-level data were mapped to gene symbols using platform-specific annotation files, and multiple probes targeting the same gene were averaged using the avereps function. Data were log2-transformed when indicated by intensity distribution, followed by quantile normalization using normalizeBetweenArrays to correct for within-array technical variation. For the GSE179285 dataset, multiple intestinal biopsies were collected from the same individuals across different anatomical sites. To eliminate pseudoreplication bias arising from treating multiple biopsies per patient as independent observations, we employed a patient-aware differential expression analysis using the duplicateCorrelation function in the limma package. Specifically, the pooled intra-patient correlation among multiple biopsies was first estimated. The resulting correlation coefficient, together with patient ID supplied to the block argument, was passed to lmFit() to adjust the effective degrees of freedom accordingly. This procedure ensures that statistical inference accounts for within-patient correlation, effectively basing significance tests on the number of independent patients rather than the total number of biopsies. DEGs were identified using limma with empirical Bayes moderation (eBayes), and P values were adjusted for multiple testing using the Benjamini-Hochberg FDR method. Genes with adjusted P value < 0.05 and |log2FC| > 0.585 were considered significant. Specifically, genes with adjusted P value < 0.05 and log2FC > 0.585 were defined as upregulated, while those with adjusted P value < 0.05 and log2FC < -0.585 were defined as downregulated. The ggplot2 package was used to create a volcano plot for visualizing DEGs, while the VennDiagram package in R software was employed to display the DEGs found in both the GSE208668 and GSE179285 datasets.Enrichment analyses for Gene Ontology (GO) functions and Kyoto Encyclopedia of Genes and Genomes (KEGG) pathways were performed on the DEGs. The analysis of GO enrichment involved biological processes (BP), cellular components (CC), and molecular functions (MF). To determine the signalling pathways connected to the DEGs, KEGG pathway enrichment analysis was carried out using the ‘clusterProfiler’ package (11). The criterion for identifying significantly enriched GO terms and KEGG pathways was set at an adjusted P value of 0.05.

### Protein–protein interaction network analysis and hub gene identification

To investigate the interactions between the common driver genes, we downloaded the gene information of the PPI networks from the Search Tool for Retrieval of Interacting Genes (STRING) database (https://string-db.org/). The interaction scores needed to be at least 0.4 to be considered significant, and the PPI network was visualized through Cytoscape software. The plugins CytoHubba and MCODE in Cytoscape were employed to identify hub genes and vital networks. In addition, the gene network of these hub genes was analysed using the GeneMANIA (https://genemania.org/).

### Animals

Adult male mice (C57BL/6, 8–10 weeks, 22–26 g) were used in the experiments for the validation of hub genes. All the mice were acquired from Shanghai Model Organisms Center, Inc. The animals were kept in cages with a stable temperature of 22 ± 0.5 °C and a relative humidity of 60% ± 2% under a 12-hour light/dark cycle that was automatically regulated, with unlimited access to food and water. All animals were anesthetized by inhalation of 1.5% isoflurane in oxygen before tissue collection. Immediately following sample harvesting, euthanasia was performed via cervical dislocation, and death was confirmed by cessation of respiration and loss of corneal reflex. All experimental protocols were approved by the Experimental Animal Ethics Committee of Fudan University.

### Establishment and identification of the mouse colitis model

The mice were split into two groups: a control group and a colitis model group, with each group containing 5 mice. From day 0 to day 7, the colitis model was initiated using 3% dextran sulfate sodium (DSS; molecular weight 36,000–50,000; MeilunBio Technology Co., Ltd., China) in the drinking water, while the control group received distilled water. The disease activity index (DAI) score, which includes three parameters—body weight, stool consistency, and faecal occult blood—was used to assess colitis severity according to a previously published grading system (12). Colon samples were collected immediately after model induction for subsequent assays.

### Sleep deprivation

The mice were divided into two groups, the control group and the sleep deprivation (SD) group, with 5 mice in each group. The mice in the SD group were subjected to sleep deprivation (from 19:00 to 13:00 of the next day) using an automated sleep deprivation system (SA109; SansBio, China) from day 1 to day 14. The automated SD system comprised a PVC cylinder, a slow motor, and a motor-driven metal bar that rotated continuously and randomly reversed direction to prevent the mice from falling asleep. Five control mice were housed in the same apparatus but could sleep normally as it was not turned on, with food and water available ad libitum. Using Ficoll–Paque PLUS (Cytiva) density gradient centrifugation (mouse peripheral blood mononuclear cell isolation solution kit; P6340; Solarbio, China), peripheral blood mononuclear cells (PBMCs) were isolated from the venous blood of mice obtained by retro-orbital bleeding after SD.

### Total RNA extraction and real-time PCR

Using TRIzol reagent (Invitrogen, USA), total RNA was isolated from the previously mentioned colon samples and PBMCs and then reverse-transcribed with a PrimeScript RT Kit (G3337; Wuhan Servicebio Technology Co., Ltd., China). A 20 μl reaction mixture was prepared for quantitative real-time PCR (qRT-PCR), which included 2 μl of cDNA, 2.5 μM forwards and reverse primers, and 7.5 μl of 2×Universal Blue SYBR Green qPCR Master Mix (G3326; Wuhan Servicebio Technology Co., Ltd., China), on a 7500 System (Applied Biosystems, USA). The number of transcripts for the target genes, including IL1B, IL7R, CCR7, ITGB2, and IRF1, was quantified using the comparative 2^−ΔΔCt^ method. The analysis of each sample was conducted in triplicate, using three independent biological replicates. The primer sequences are listed in **Table 1**.

**Table 1:** Primer sequences for qRT-PCR valizdation of hub genes.

Gene	Forward primer sequence (5’->3’)	Reverse primer sequence (5’->3’)
IL1B	GCATCCAGCTTCAAATCTCGC	TGTTCATCTCGGAGCCTGTAGTG
CCR7	GAGGCTCAAGACCATGACGGA	ATCCAGGACTTGGCTTCGCT
ITGB2	GCCGCATTCAATGTGACTTTC	TTGGGACATGGGTTCCTCAG
IL7R	CTGAAAGTCGTTTATCGCAAAGAAG	TTCTCTGTGGGATTGTTGTTCTTG
IRF1	CTCTACCCTACCTGATGACCACA	TCCTCGTCTGTTGCGGCTT

### Enzyme-linked immunosorbent assay

PBMCs were washed with phosphate-buffered saline (PBS), resuspended in ice-cold lysis buffer at approximately 1 × 10^6^ cells/mL, and lysed on ice for 30 min. Colon tissues were accurately weighed and homogenized in ice-cold PBS at a ratio of 1:9 (w/v). Tissue homogenates and cell lysates were centrifuged at 2,000–3,000 rpm for 20 min at 4 °C, and the supernatants were carefully collected. Total protein concentrations were determined using a BCA protein assay kit for normalization. The protein levels of IL-1β, IRF1, ITGB2, CCR7, and IL-7R in colon tissue homogenates and PBMC lysates were quantified using commercial ELISA kits according to the manufacturer’s instructions.

### Prediction of the TF–miRNA–mRNA regulatory network

To explore putative regulatory mechanisms of the hub genes, we performed an in silico analysis of transcription factors (TFs) and microRNAs (miRNAs). TF–target interactions were retrieved from TRRUST (Transcriptional Regulatory Relationships Unraveled by Sentence-based Text mining, http://www.grnpedia.org/trrust), a database of predicted transcriptional regulatory relationships (13). Furthermore, miRNA–target interactions were sourced from miRWalk (http://mirwalk.umm.uni-heidelberg.de/), a publicly accessible database of predicted miRNA–target interactions. These computational predictions were integrated and visualized as a putative TF–miRNA–mRNA regulatory network using Cytoscape

### Correlation between immune infiltration and shared hub genes

To assess immune cell infiltration, we employed three transcriptome deconvolution algorithms: CIBERSORT (Cell-type Identification By Estimating Relative Subsets Of RNA Transcripts) (14), which estimates the relative proportions of 22 immune cell subsets based on the LM22 signature matrix; EPIC (Estimating the Proportions of Immune and Cancer cells) (15), which quantifies fractions of immune, stromal and malignant cells via constrained least squares regression; and xCell (16), which computes enrichment scores for 64 distinct cell populations through single-sample gene set enrichment analysis (ssGSEA). All three algorithms were applied to transcriptomic profiles of both healthy control and disease cohorts. In addition, Spearman’s correlation analysis was performed to evaluate pairwise correlations between shared hub genes and the infiltration levels of diverse immune cell populations.

### Prediction of potential drug candidates

The exploration of protein–drug interactions is crucial for screening potential therapeutic targets for insomnia and IBD. The validated common hub genes of insomnia and IBD were entered into the Enrichr platform (https://maayanlab.cloud/Enrichr/). We subsequently screened drug candidates related to the hub genes using the Drug Signatures Database (DSigDB) (17). Finally, predicted drug–target interactions were integrated to construct a predictive drug–mRNA network with Cytoscape.

### Statistical analysis

Statistical analysis was performed using R language (version 4.2.2) and GraphPad Prism 10. The statistical significance between the two groups was evaluated using Student’s t test, with significance levels set at *P < 0.05, **P < 0.01, and ***P < 0.001.

## Results

### Identification of common DEGs between insomnia and IBD

**Figure 1:**
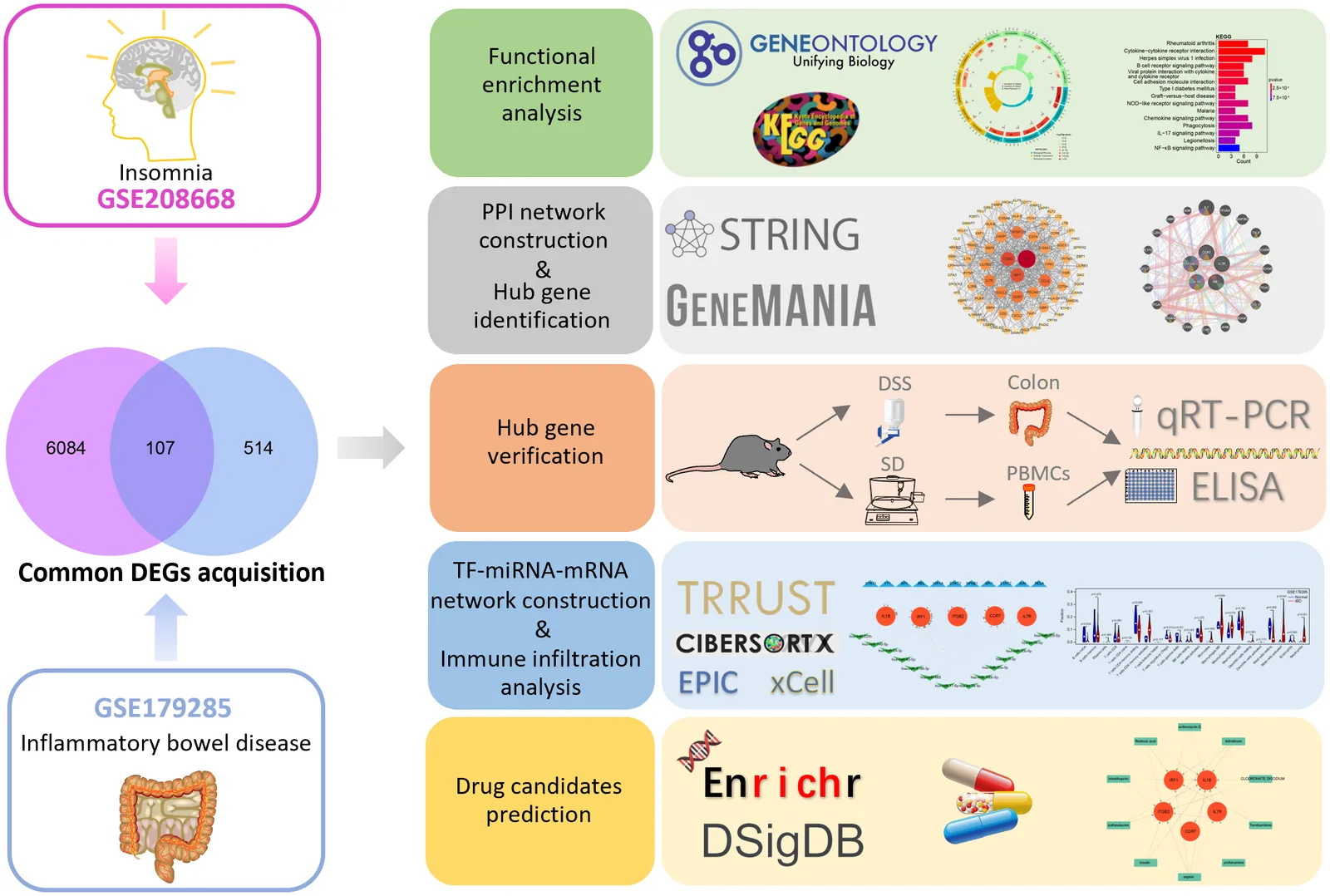
Research flowchart. Samples from two categories, insomnia and inflammatory bowel disease, were gathered from the GSE208668 and GSE179285 datasets, respectively. The DEGs shared between the two datasets were identified. Functional enrichment, PPI network construction, hub gene identification, hub gene verification, TF–miRNA–mRNA network construction, immune infiltration analysis, and drug candidate prediction were performed on the common DEGs. TF, transcription factor; DEGs, differentially expressed genes; PPI, protein–protein interaction.

**Figure 2:**
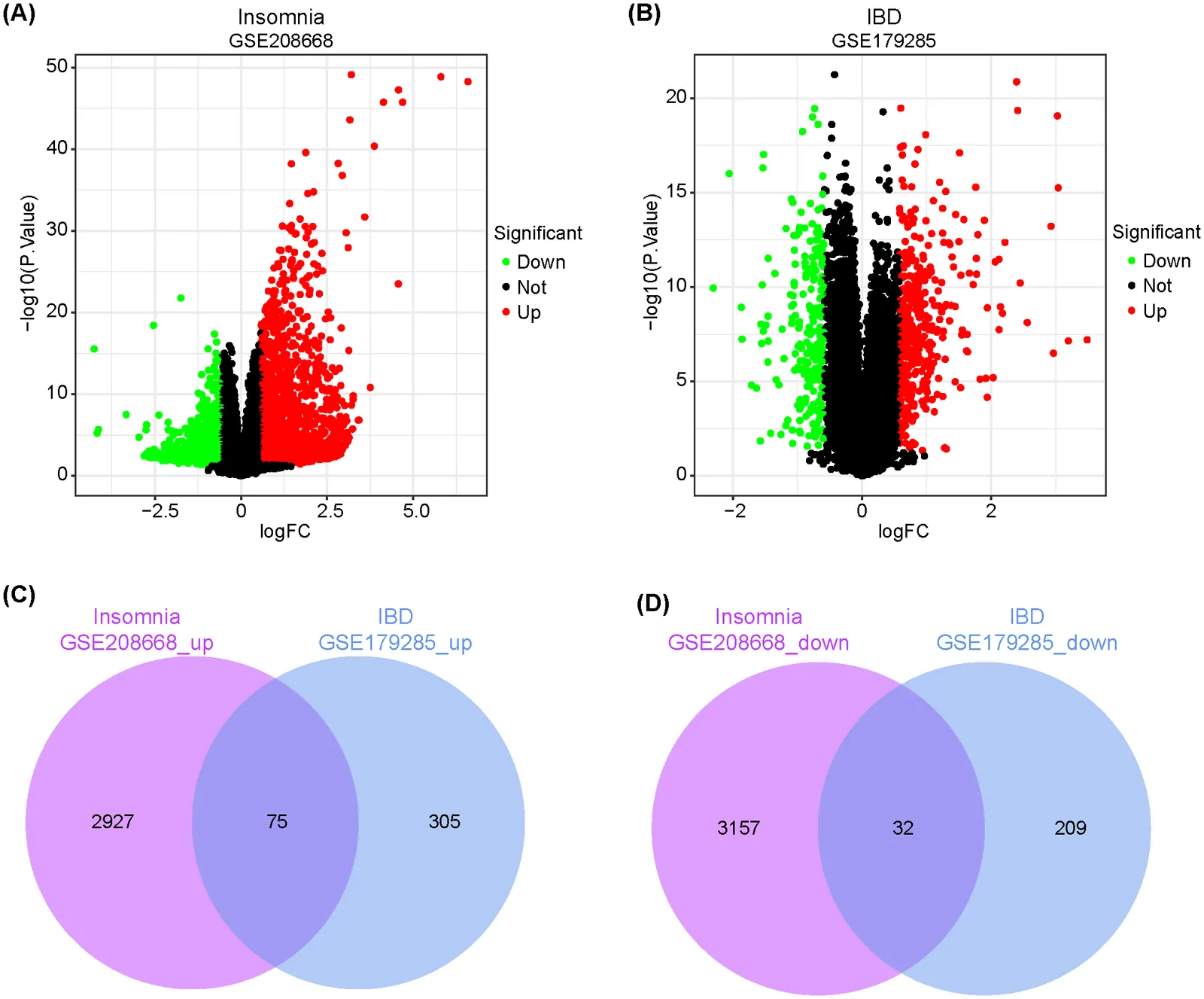
DEGs common to insomnia and inflammatory bowel disease. Volcano plot of DEGs in the insomnia-related GSE208668 dataset **(A)** and IBD-related GSE179285 dataset **(B)**. Upregulated DEGs are marked by red dots, downregulated DEGs are marked by blue dots, and genes with no significant differences between the groups are marked by grey dots. Common upregulated **(C)** and downregulated **(D)** DEGs shared between the GSE208668 dataset (depicted in purple) and the GSE179285 dataset (depicted in blue) are illustrated using Venn diagrams. In the overlap of these two datasets, there are 107 DEGs, comprising 75 upregulated genes and 32 downregulated genes. DEGs, differentially expressed genes; IBD, inflammatory bowel disease.

This study’s workflow is shown in **Figure 1**. To explore the interaction and crosstalk between insomnia and IBD, we obtained and analysed the GSE208668 and GSE179285 datasets from the GEO database. Compared with genes in the control group, genes whose adjusted P value < 0.05 were defined as DEGs; those whose FC was > 1.5 were considered upregulated DEGs, whereas those whose FC was < –1.5 were considered downregulated DEGs. The GSE208668 and GSE179285 datasets were used to identify DEGs associated with insomnia and IBD, respectively. Using the GSE208668 insomnia dataset, 6191 DEGs were discovered, comprising 3002 upregulated genes and 3189 downregulated genes (**Figure 2A**). From the GSE179285 IBD dataset, 621 DEGs were identified, including 380 upregulated and 241 downregulated genes (**Figure 2B**). Among the DEGs from the insomnia and IBD datasets, we identified 75 genes that were upregulated in both datasets and 32 that were downregulated in both datasets (**Figures 2C, D**).

### Functional enrichment analysis of common DEGs

**Figure 3:**
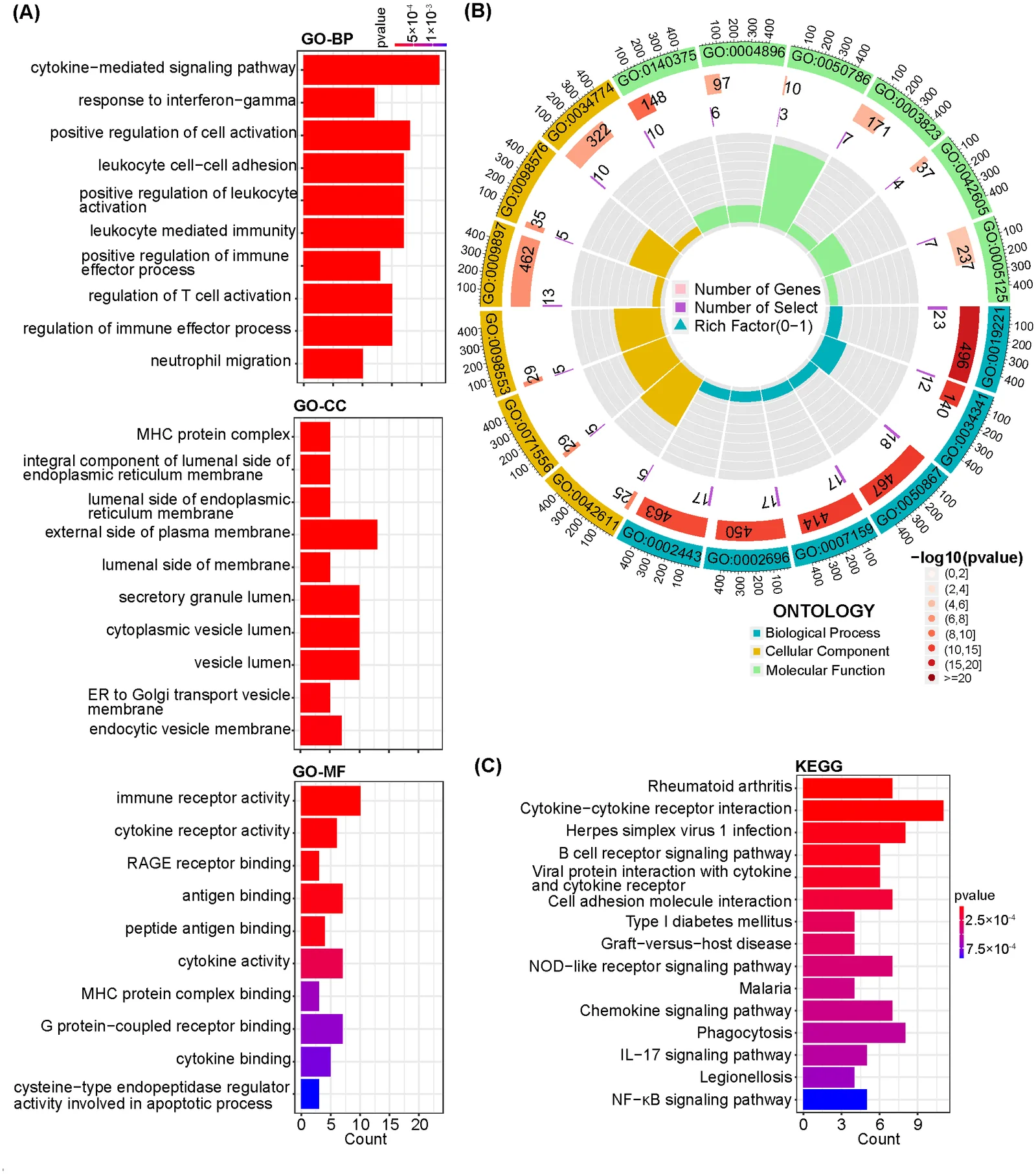
Functional enrichment analysis of common DEGs between the two groups. Bar chart **(A)** and circle diagram **(B)** display the GO enrichment results (biological process, cellular component, molecular function) for the 107 DEGs shared between insomnia and IBD. **(C)** KEGG pathway enrichment of the same 107 DEGs. GO, Gene Ontology; BP, biological process; MF, molecular function; CC, cellular component; KEGG, Kyoto Encyclopedia of Genes and Genomes.

To investigate the biological roles and pathways of the overlapping genes from the insomnia and IBD datasets, we carried out GO enrichment and KEGG pathway analyses on the common DEGs. In the BP category, these common DEGs were involved primarily in cytokine-mediated signalling pathway, response to interferon-gamma, positive regulation of cell activation, leukocyte cell–cell adhesion, and positive regulation of leukocyte activation (**Figure 3A**). In the CC category, these common DEGs were primarily localized to the MHC protein complex, integral component of lumenal side of endoplasmic reticulum membrane, the lumenal side of endoplasmic reticulum membrane, the external side of plasma membrane, and the lumenal side of membrane, among others (**Figure 3A**). In terms of MF, these common DEGs were predominantly associated with immune receptor activity, cytokine receptor activity, RAGE receptor binding, antigen binding, and peptide antigen binding, among others (**Figure 3A**). The numbers of genes within the top 6 most enriched GO terms of the DEGs are displayed in the circular diagram for BP, CC and MF (**Figure 3B**). KEGG analysis revealed that these common DEGs were involved in rheumatoid arthritis, cytokine–cytokine receptor interaction, cell adhesion molecule interaction, malaria, the B-cell receptor signalling pathway, herpes simplex virus 1 infection, viral protein interaction with cytokine and cytokine receptor, type 1 diabetes mellitus, graft-versus-host disease, the NOD-like receptor signalling pathway, the chemokine signalling pathway, the IL-17 signalling pathway, legionellosis, the NF-κB signalling pathway, and phagocytosis (**Figure 3C**). Our findings suggest that the inflammatory response and immune activation are pivotal drivers underlying the pathogenesis of both insomnia and IBD.

### PPI network analysis and hub gene identification

**Figure 4:**
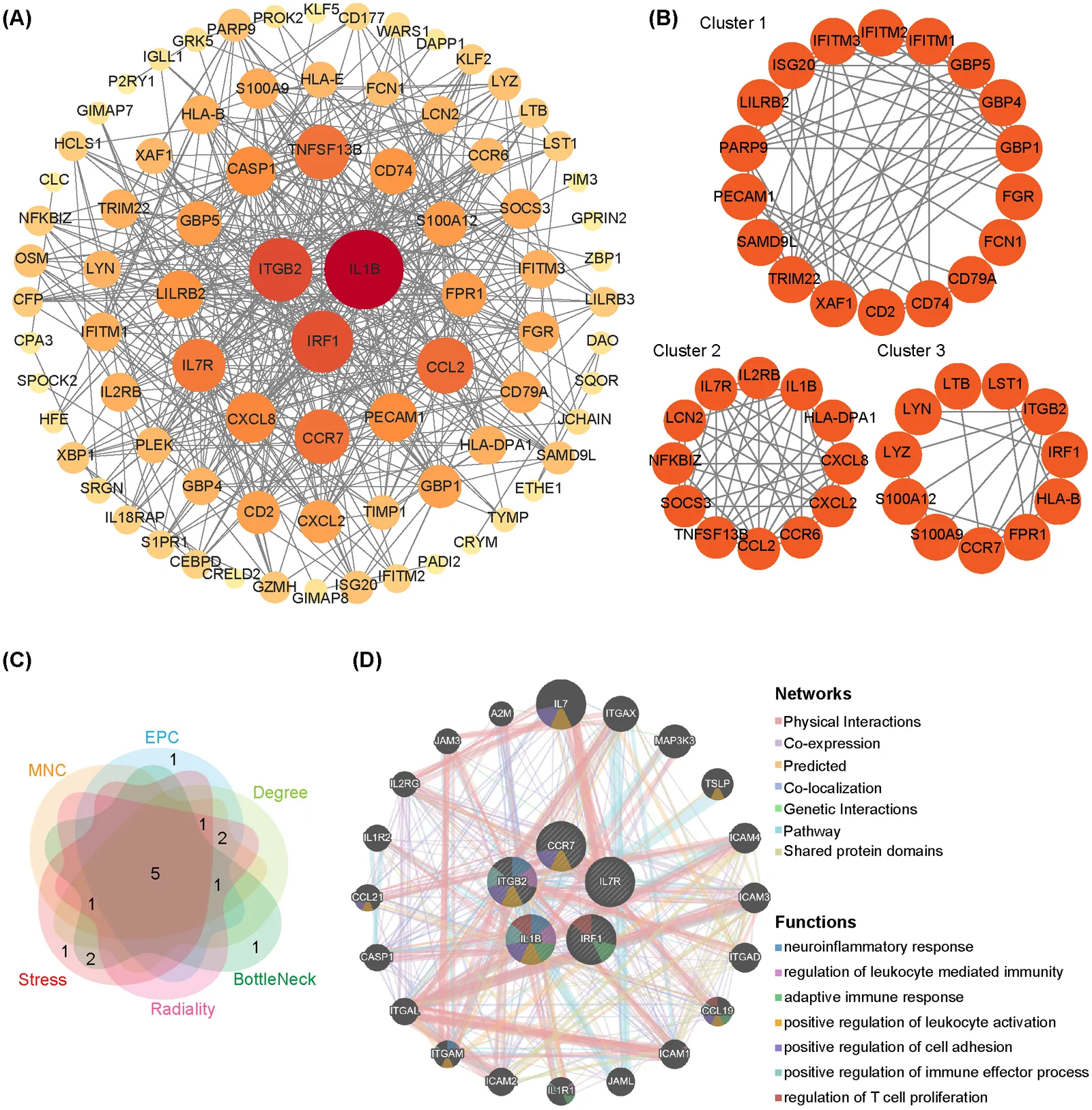
Protein–protein interaction network analysis, hub gene identification and functional enrichment. **(A)** The PPI network of common DEGs shared by insomnia and inflammatory bowel disease is depicted, with red node colour intensity indicating the strength of connectivity with other genes. **(B)** Three cluster modules extracted by the MCODE plugin in Cytoscape. **(C)** The 5 hub genes were identified using the CytoHubba plugin in Cytoscape, which is based on six algorithms: EPC, Degree, BottleNeck, Radiality, Stress, and MNC. **(D)** GeneMANIA was used to construct a gene network and perform functional analysis of the five hub genes. The hub genes are shown in the inner circle, while the outer circle displays their related reciprocal genes. The colours of the nodes represent gene function annotations, including those related to the neuroinflammatory response, regulation of leukocyte-mediated immunity, adaptive immune response, positive regulation of leukocyte activation, positive regulation of cell adhesion, positive regulation of immune effector processes, and regulation of T-cell proliferation. The colours of the edges indicate the type of interaction based on physical interactions, coexpression, predicted interactions, colocalization, genetic interactions, pathways and shared protein domains. PPI, protein–protein interaction; DEGs, differentially expressed genes; MCODE, molecular complex detection; MNC, maximum neighborhood component; EPC, edge percolated component.

To map the PPI network among the 107 shared DEGs, the list was first submitted to STRING; the generated file was then imported into Cytoscape for visualization (**Figure 4A**). The MCODE plugin was applied to identify key clusters and recognize three modules: Cluster 1 (18 nodes, 62 edges, score=7.294), including IFITM2, IFITM3, IFITM1, ISG20, GBP5, LILRB2, GBP4, PARP9, GBP1, PECAM1, FGR, FCN1, SAMD9L, TRIM22, CD79A, XAF1, CD74, and CD2; Cluster 2 (12 nodes, 43 edges, score=7.818), including IL2RB, IL1B, IL7R, LCN2, HLA-DPA1, CXCL8, NFKBIZ, SOCS3, CXCL2, TNFSF13B, CCL2, and CCR6; and Cluster 3 (11 nodes, 22 edges, score=4.400), including LTB, LST1, LYN, ITGB2, LYZ, IRF1, S100A12, HLA-B, S100A9, CCR7, and FPR1 (**Figure 4B**).

Using the CytoHubba plug-in, hub genes were identified from the PPI network through six different algorithms: Degree, BottleNeck, Stress, Radiality, Maximum Neighborhood Component (MNC) and Edge Percolated Component (EPC) (**Table 2**). After intersection, five genes were considered the core targets of insomnia and IBD (**Figure 4C**). GeneMANIA was used to construct the gene network and analyse the coexpression network and related functions of these genes. These genes were associated with a complex PPI network, comprising 77.54% physical interactions, 8.01% coexpression, 5.37% predicted interactions, 3.63% colocalization, 2.87% genetic interactions, 1.88% pathway interactions, and 0.60% shared protein domains (**Figure 4D**). Gene functional annotation revealed that these hub genes are closely related to immune responses and inflammatory processes, encompassing three major aspects: neuroinflammatory responses bridging the central and peripheral systems; activation and regulation of adaptive immunity, particularly leukocyte-mediated immunity and T-cell proliferation; and enhanced leukocyte activation, cell adhesion capacity, and immune effector processes (**Figure 4D**).

**Table 2:** The top 10 hub genes analyzed by cytoHubba.

Degree	EPC	BottleNeck	Radiality	Stress	MNC
IL1B ITGB2 IRF1 CCL2 CCR7 TNFSF13B IL7R CXCL8 CASP1 PECAM1	IL1B ITGB2 CCL2 IRF1 IL7R CCR7 TNFSF13B PECAM1 CXCL8 CD74	IL1B ITGB2 PECAM1 CD2 CCR7 CASP1 SQOR IRF1 IL7R FGR	IL1B ITGB2 IRF1 TNFSF13B CCR7 CCL2 PECAM1 IL7R CASP1 CXCL8	IL1B CASP1 IRF1 ITGB2 CCR7 SQOR TNFSF13B LILRB2 FGR IL7R	IL1B ITGB2 IRF1 CCL2 CCR7 TNFSF13B IL7R CXCL8 PECAM1 CASP1

Degree, EPC, BottleNeck, Radiality, Stress and MNC represent different algorithms of CytoHubba plugin in Cytoscape.

### Validation of hub gene expression by qRT–PCR and ELISA

**Figure 5:**
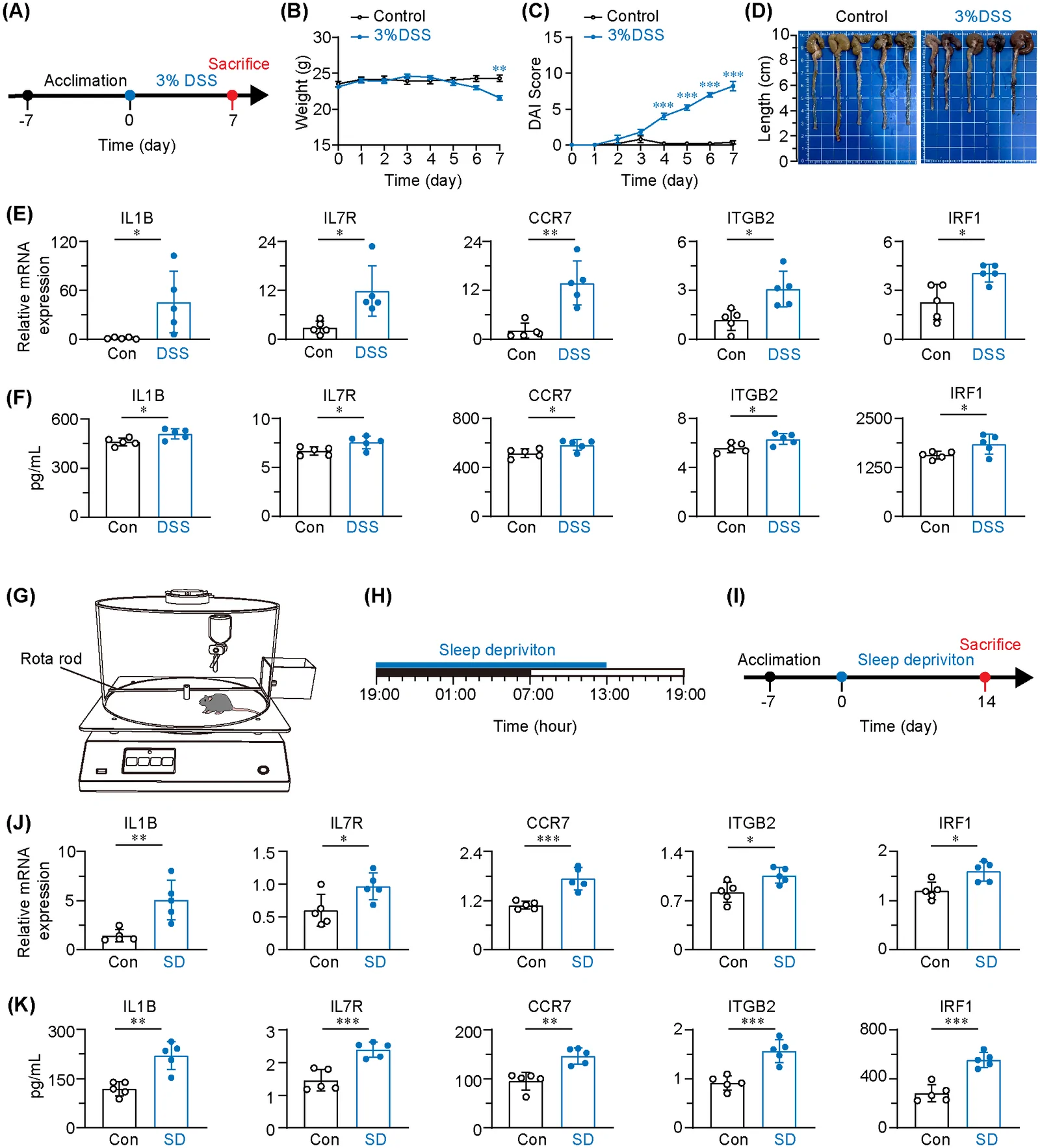
Verification of the hub genes by qRT-PCR and ELISA. **(A)** Schematic diagram of the mouse model of colitis. **(B)** Body weights of the two groups of mice from 0 to 7 days. **(C)** DAI scores of the two groups of mice from 0 to 7 days. **(D)** Representative images of colons from the two groups of mice. **(E)** Validation of the differential mRNA expression of five key genes in the colons of the two groups of mice using qRT–PCR. **(F)** Validation of the differential protein expression of five key genes in the colons of the two groups of mice using ELISA. **(G)** Schematic diagram of the sleep deprivation system. **(H)** Schematic diagram of sleep deprivation for one day. **(I)** Schematic diagram of sleep deprivation for 14 days. **(J)** Validation of the differential mRNA expression of five key genes in the PBMCs of the two groups of mice using qRT–PCR. **(K)** Validation of the differential protein expression of five key genes in the PBMCs of the two groups of mice using ELISA. *P < 0.05; **P < 0.01; ***P < 0.001.

To verify the reliability of the five hub genes, we quantified their expression by qRT–PCR and ELISA in both colitis and insomnia mouse models. Compared with normal mice, DSS-induced colitis model mice showed decreased body weight and colon length, but increased DAI scores, confirming successful model establishment (**Figures 5A–D**). All the hub genes were markedly upregulated in the colon relative to those in the controls (**Figures 5E, F**). Moreover, in a mouse model of insomnia induced by SD for 14 consecutive days, the expression levels of IL1B, IL7R, CCR7, ITGB2, and IRF1 in PBMCs increased significantly (**Figures 5G–K**). Collectively, the results confirmed the dysregulation of these five hub genes—IL1B, IL7R, CCR7, ITGB2 and IRF1—in both disease conditions.

### TF–miRNA–mRNA regulatory network construction

**Figure 6:**
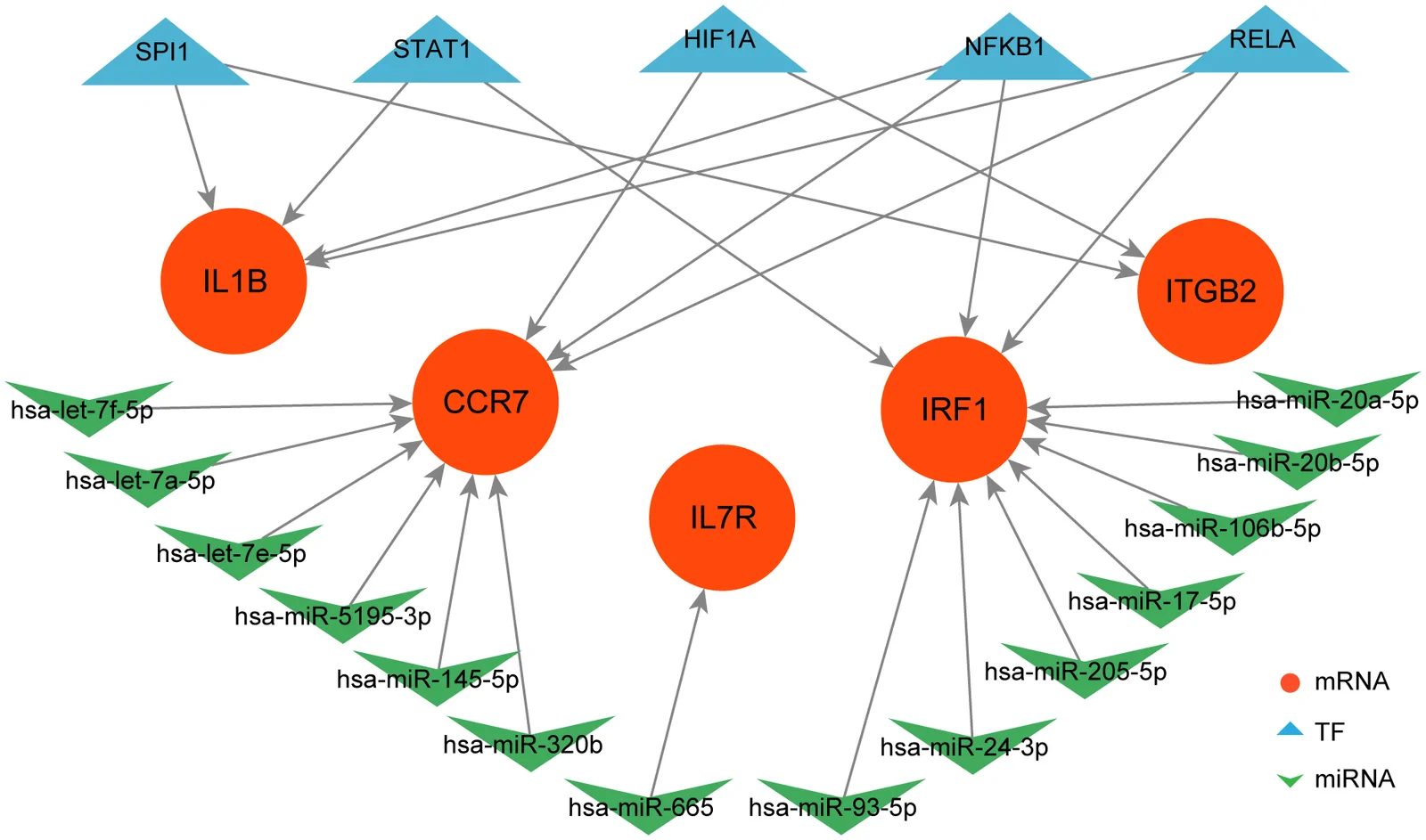
The predicted TF–miRNA–mRNA regulatory network. Red circles represent hub genes, blue triangles represent TFs that target hub genes, and inverted green triangles represent miRNAs. TF, transcription factor.

To elucidate the putative regulatory interactions, we constructed a comprehensive TF–miRNA–mRNA network of the hub genes using Cytoscape. TF–mRNA interactions were obtained from the TRRUST database, while miRNA–mRNA relationships were predicted using miRWalk. A network comprising 24 nodes (5 TFs, 14 miRNAs, and 5 mRNAs) and 26 interactions was constructed (**Figure 6**). Specifically, the network predicted the following potential enrichment for TFs targeting the hub genes: IL1B with RELA, NF-κB1, SPI1, and STAT1; CCR7 with RELA, NF-κB1, and HIF1A; IRF1 with RELA, NF-κB1, and STAT1; and ITGB2 with HIF1A and SPI1 (**Figure 6**). The identified associations suggest a potential regulatory network in which specific TFs may modulate the expression of the hub genes, thereby providing a framework for understanding their transcriptional dynamics during insomnia and IBD. Among the miRNAs, CCR7 and IRF1 exhibited the greatest connectivity, with predicted coexpressing relationships with 6 and 7 miRNAs, respectively. IL7R was predicted to be coexpressed with only one miRNA, whereas ITGB2 and IL1B were not predicted to be coexpressed with any other miRNAs. Taken together, these results provide a computational basis for exploring the complex molecular interplay between IBD and insomnia, thereby highlighting candidate interactions for future experimental validation in subsequent studies.

### Immune cell infiltration and its correlation with shared hub genes

**Figure 7:**
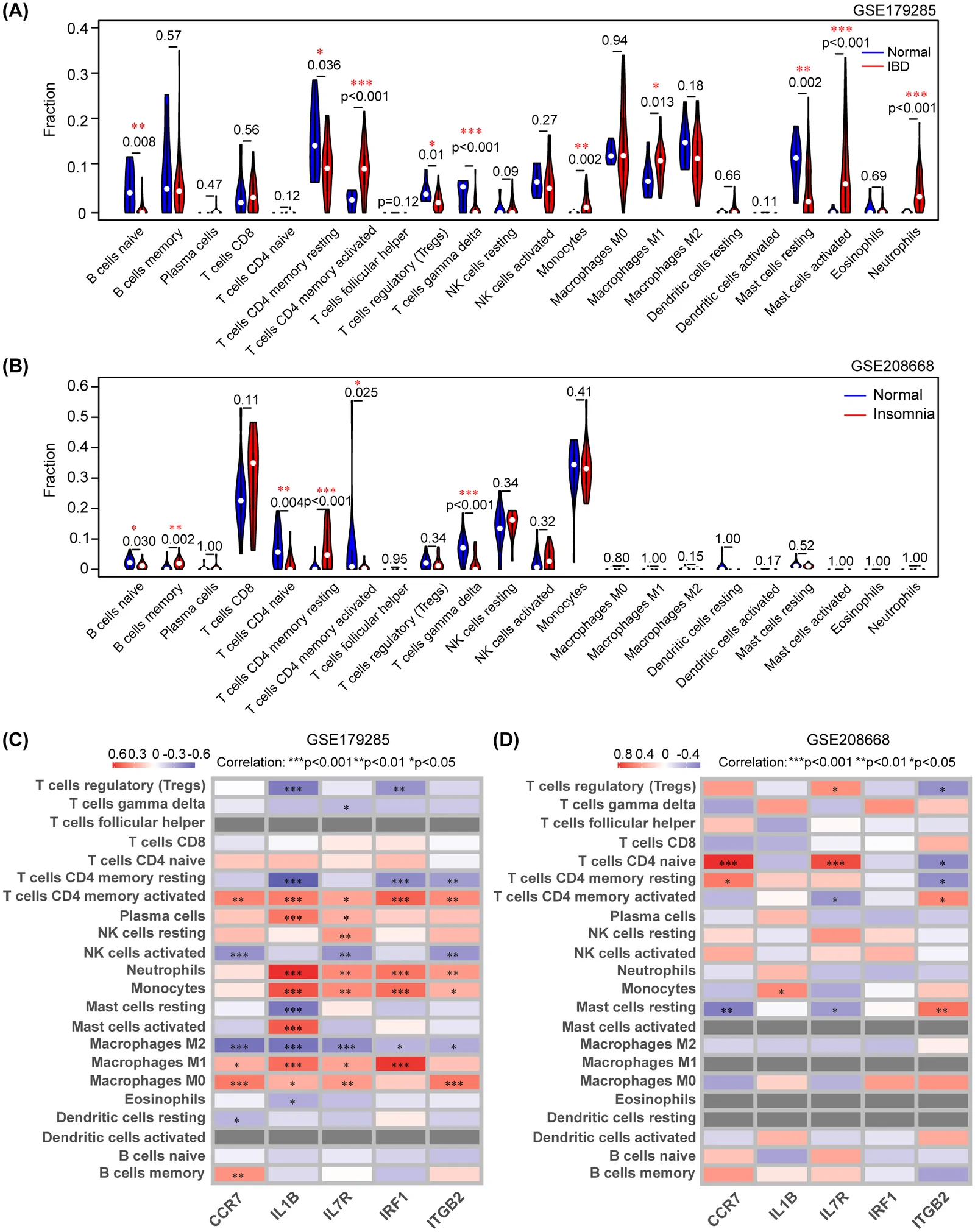
CIBERSORT immune infiltration profiling and hub gene–immune correlation analysis in IBD and insomnia cohorts. **(A, B)** Violin plots illustrating the immune cell infiltration patterns in the IBD-GSE179285 and insomnia-GSE208668 cohorts, with blue indicating normal individuals and red indicating IBD/insomnia patients. **(C, D)** Heatmaps illustrating the correlation between hub genes and immune cells in the IBD-GSE179285 cohort and the insomnia-GSE208668 cohort. Positive correlations are represented by red, and negative correlations are represented by blue. *P < 0.05; **P < 0.01; ***P < 0.001.

To dissect the immune landscape of IBD and insomnia, we profiled both datasets using three independent transcriptomic deconvolution algorithms: CIBERSORT, which quantifies 22 immune cell subsets (**Figures 7A, B**); EPIC, which characterizes seven major immune and stromal cell populations (**Supplementary Figures 1A, B**); and xCell, which annotates 64 distinct cell populations, of which only 25 representative immune subtypes were presented for clarity and conciseness (**Supplementary Figures 2A, B**). In the IBD dataset, consistent results across algorithms revealed prominent immune cell alterations. All three algorithms collectively confirmed a significant increase in macrophage abundance. CIBERSORT and xCell detected a significant depletion of naïve B cells and a marked expansion of neutrophils. Additionally, CIBERSORT identified elevated activated memory CD4^+^ T cells, while xCell and EPIC further verified increased CD8^+^ T cell levels. These immune perturbations indicate a pro-inflammatory profile in IBD, driven by robust innate immune activation and adaptive immune dysregulation. The insomnia dataset exhibited a distinct yet partially overlapping immune signature relative to IBD. CIBERSORT analysis revealed increased memory B cells and resting memory CD4^+^ T cells, with concurrent decreases in naïve B cells, naïve CD4^+^ T cells, activated memory CD4^+^ T cells, and γδ T cells. EPIC confirmed a significant reduction in CD8^+^ T cells, whereas xCell revealed upregulated Tregs and downregulated Th2 cells. Despite identifying different T cell subsets, all three algorithms pointed to T cell compartment disruption as a hallmark of insomnia. Cross-disease comparison revealed shared mechanisms of T cell dysregulation. CIBERSORT identified decreased γδ T cells in both conditions, suggesting a conserved loss of innate-like lymphocytes. EPIC further showed reduced CD8^+^ T cells across both datasets. In xCell, distinct T cell subsets exhibited divergent dysregulation patterns in both diseases. Together, these findings establish T cell disturbances as a shared immunological feature of IBD and insomnia, despite their distinct primary pathologies.

Using the immune cell infiltration estimates derived from CIBERSORT, EPIC, and xCell, we performed correlation analyses between hub gene expression and immune cell levels. In the IBD dataset, all five hub genes showed positive correlations with CD4^+^ T cell levels, with consistent results across the three deconvolution methods (**Figure 7C**; **Supplementary Figures 1C**, **2C**). Additionally, IL1B, IL7R, IRF1, and ITGB2 were positively associated with neutrophil and monocyte levels based on CIBERSORT and xCell estimates (**Figure 7C**; **Supplementary Figure 2C**). B cell levels correlated positively with CCR7, IL7R, and ITGB2 in EPIC and xCell, whereas CD8^+^ T cell levels showed negative associations with IL1B and IRF1 in these two methods (**Supplementary Figures 1C**, **2C**). In the insomnia dataset, IL1B, ITGB2, CCR7, and IL7R exhibited immune infiltration correlations partially concordant with those in IBD. IL1B maintained positive correlations with monocyte levels in CIBERSORT and xCell (**Figure 7D**; **Supplementary Figure 2D**). ITGB2 recapitulated the CIBERSORT-derived pattern observed in IBD, showing positive associations with activated memory CD4^+^ T cells and negative associations with resting memory CD4^+^ T cells (**Figure 7D**). Most notably, CCR7 and IL7R displayed significant positive correlations with CD4^+^ T cell levels across all three deconvolution methods (**Figure 7D**; **Supplementary Figures 1D**, **2D**), suggesting that CD4^+^ T cell–mediated immune regulation represents a key molecular mechanism underlying IBD–insomnia comorbidity. Collectively, these findings—derived from correlation analyses of immune infiltration estimates by multiple deconvolution methods—suggest that the five hub genes reshape immune microenvironment composition by modulating CD4^+^ T cell subsets, macrophages, monocytes, and neutrophils. Conserved associations between IBD and insomnia reveal shared immune mechanisms, whereas disease-specific divergences reflect distinct pathological features.

### Prediction of candidate drugs on the basis of hub genes

**Figure 8:**
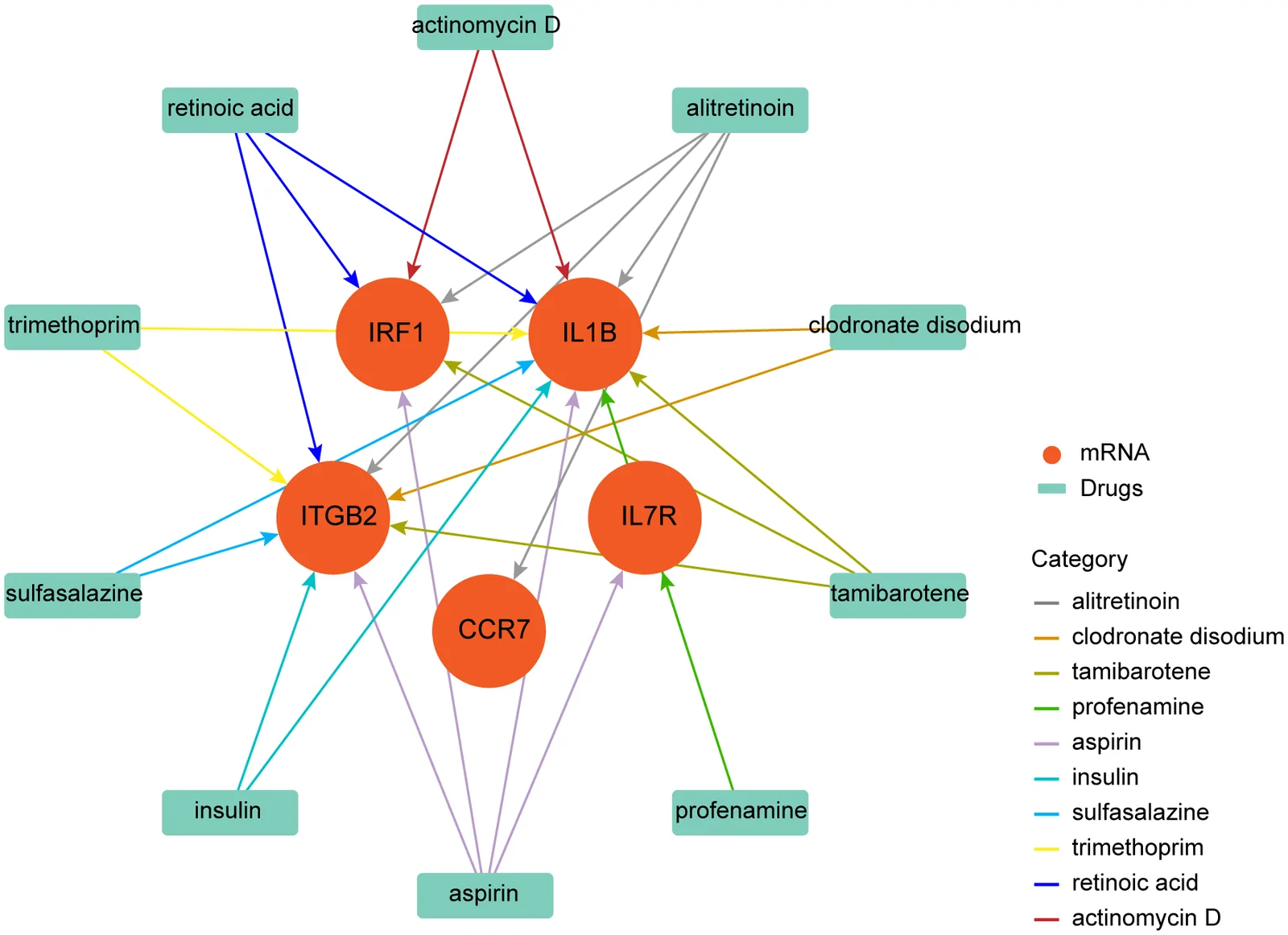
The predicted drug–mRNA interaction network of the hub genes. Red circles represent hub genes, and green rounded rectangles represent candidate drugs.

To explore putative therapeutics for insomnia and IBD, we constructed a drug–mRNA interaction network based on DSigDB using five hub genes as the molecular signature. A total of 612 compounds were predicted as targeting the hub genes, among which 86 were predicted to target more than two hub genes (**Supplementary File 2**). We presented the top 10 compounds that were predicted to target more than two hub genes and are listed as drugs in public databases, including alitretinoin, aspirin, clodronate disodium, retinoic acid, sulfasalazine, profenamine, tamibarotene, actinomycin D, trimethoprim, and insulin (**Figure 8**). Among these 10 drugs, two were predicted to target four hub genes—alitretinoin was predicted to target IL1B, IRF1, ITGB2, and CCR7, whereas aspirin was predicted to target IL1B, IRF1, ITGB2, and IL7R. These predictions are entirely computational and require extensive experimental validation, including *in vitro* binding assays, cell-based functional studies, and preclinical pharmacological evaluation, before any therapeutic potential can be assessed. Other influencing factors still require evaluation, necessitating further investigation.

## Discussion

There is a significant and complex bidirectional interaction between IBD and insomnia. IBD drives the onset of insomnia through multiple pathways, while insomnia, in turn, exacerbates IBD by promoting a systemic low-grade inflammatory state, lowering pain thresholds, and impairing treatment adherence, thereby potentially intensifying intestinal inflammation and triggering disease relapse. Therefore, elucidating shared molecular signatures connecting the two disorders is critical to provide a basis for improved diagnostic and therapeutic strategies.

In the present study, we identified 107 overlapping DEGs between insomnia and IBD, comprising 75 upregulated and 32 downregulated genes. Functional enrichment analysis revealed that immune activation and inflammatory responses represent the core pathophysiological hallmarks of both conditions. Through PPI network construction, we identified five hub genes—IL1B, IL7R, CCR7, ITGB2, and IRF1—which were subsequently validated in insomnia and UC mouse models. These genes play pivotal roles in immune regulation and may mediate the comorbidity between insomnia and IBD. Furthermore, we elucidated the TF and miRNA regulatory networks governing the transcriptional and posttranscriptional control of these hub genes. Additionally, we characterized immune cell infiltration patterns in both diseases and investigated their associations with hub gene expression. Finally, the DSigDB was utilized to predict potential therapeutic agents targeting these hub genes for concurrent modulation of immune dysregulation in insomnia and IBD. Collectively, our findings reveal shared molecular signatures and potential pathogenic pathways associated with the two disorders, providing candidate molecular targets and a mechanistic basis for exploring combined therapeutic strategies.

Notably, our findings indicate that inflammation and immune system dysregulation play significant roles in the occurrence and progression of both IBD and insomnia. The overlapping DEGs screened in this study were derived from two distinct tissue sources—peripheral blood for insomnia and intestinal tissue for IBD—yet they converge on markers of systemic inflammatory activation rather than tissue-specific pathogenic signals. These five genes constitute a comprehensive axis from inflammatory initiation to immune cell response. IL1B functions as a core circulating pro-inflammatory cytokine capable of crossing the blood-brain barrier to disrupt sleep architecture and simultaneously exacerbate intestinal mucosal inflammatory infiltration (18–20). ITGB2, CCR7 and IL7R regulate the systemic trafficking of immune lymphocytes between peripheral circulation, gut lymphoid tissue and central immune microenvironment, orchestrating widespread immune activation beyond local intestinal tissue responses (21–24). IRF1 acts as a universal inflammatory transcription factor triggering conserved inflammatory transcriptional programs in both circulating and mucosal immune cells (25). Collectively, the overlapping DEGs identified herein capture a convergent systemic inflammatory response axis linking intestinal chronic inflammation and sleep impairment, rather than tissue-restricted molecular mechanisms unique to intestinal or brain tissue.

IL1B, the most extensively studied member of the IL-1 family, is a potent proinflammatory cytokine produced primarily by innate immune cells such as monocytes, macrophages, and dendritic cells (18, 26). As one of the earliest and most powerful mediators of inflammatory responses, it functions as the “alarmin” of inflammation. In this study, we identified IL1B as the core gene with the highest score across the four algorithms for the comorbidity of IBD and insomnia. Prior studies of adults have revealed associations between IL1B and restless legs syndrome (20), daytime fatigue (27) and sleep onset insomnia (28). Numerous studies have also shown that IL1B is closely associated with sleep regulation (29). Consistent with our findings, sleep deprivation can increase IL1B expression in the brain, peripheral tissue, and circulation in many species, including rabbits, cats, monkeys, mice, rats, and humans (29). Moreover, it is well known that IL1B is a key inflammatory mediator in the development and progression of IBD, and its expression level is closely correlated with disease severity (30, 31). In addition, genetic and pharmacological blockade of IL1B has been shown to attenuate experimental colitis (19). Our study further supports the vital involvement of IL1B in IBD and insomnia, suggesting that targeting IL1B may be a potential therapeutic strategy for IBD patients with insomnia symptoms.

IRF1 is a transcription factor that acts as a master regulator of innate immunity, apoptosis and tumour suppression (32). It transcriptionally activates interferon genes, proapoptotic targets and cell cycle checkpoints, thereby exerting antiviral, antitumour and immunoregulatory functions, which are closely associated with the pathogenesis of IBD (32). Integrative multi-omic and mendelian randomization studies have established IRF1 as a causal, diagnostic and druggable hub gene in IBD (33). In a DSS-induced murine colitis model as well as in human IBD intestinal mucosa, both IRF1 mRNA and protein levels are significantly elevated (32, 34). On the basis of the results of an integrative bioinformatics analysis, this study identified IRF1 as a shared hub gene bridging IBD and insomnia, whose dual pathogenic roles were further validated through animal experimental models. Our findings concerning IBD are consistent with those in the literature, confirming the central role of IRF1 in regulating intestinal inflammation. We also found that IRF1 is highly expressed in PBMCs from individuals with insomnia, and this finding was validated in an SD mouse model. This is an innovative finding, given that research on IRF1 and sleep remains scarce, our study provides a valuable theoretical reference.

IL-7R is a heterodimeric complex composed of an IL-7Rα chain and a γ chain (35). The signalling pathway formed by IL-7R and its ligand IL-7 plays a crucial role in the immune system, particularly in lymphocyte development, survival, and homeostasis maintenance (36, 37). Dysregulation of the IL-7R signalling pathway is closely associated with autoimmune diseases, such as multiple sclerosis, IBD, type 1 diabetes, rheumatoid arthritis, and atopic dermatitis (38, 39). Overexpression of the IL-7R signalling pathway is a key mechanism of treatment failure in IBD, highlighting its dual value as a core therapeutic target and potential biomarker and providing a new direction for addressing the unmet needs in clinical IBD diagnosis and treatment (36). IL7R remains understudied in insomnia pathogenesis, with limited evidence supporting its involvement (40). This study conducts a transcriptome-based analysis integrating datasets of IBD and insomnia, establishing IL7R as a pivotal factor in both conditions and validating its involvement through the use of animal models. While the role of IL7R in IBD has been extensively studied, its involvement in insomnia pathophysiology requires further investigation.

CCR7, a CC-chemokine receptor for CCL19 and CCL21 expressed by various immune cell subsets, is a key chemokine receptor crucial for lymphocyte trafficking and immune homeostasis (41, 42). Crohn’s-like murine ileitis studies have shown that CCR7 deficiency causes abnormal retention of pathogenic Th1 effector T cells in the gut and simultaneously impairs the generation and homing of regulatory T cells, thereby disrupting the Th1/Th17/Treg balance and aggravating tissue damage (43). Conversely, in active human CD lesions, large numbers of CCR7^+^LAMP3^+^ mature dendritic cells accumulate around T-cell clusters, suggesting that these cells are trapped in the mucosa, where they may sustain or amplify inflammation (44). These previous studies collectively revealed that targeting the CCR7 pathway to regulate immune cell migration may represent a potential therapeutic strategy for IBD. Although direct evidence implicating CCR7 itself in insomnia mechanisms remains limited—as large cross-sectional studies have primarily employed it merely as a surface marker for naïve T cells (CD8^+^CD45RA^+^CCR7^+^) without assessing its specific expression or functional roles in sleep—reduced levels of these CCR7-positive naïve T cells are significantly associated with difficulty falling asleep and short sleep duration (45). Notably, we observed upregulated CCR7 expression in PBMCs from insomnia patients and colon tissues from IBD patients, and these findings were validated in mouse models of colitis and SD. These findings suggest that dysregulation of the CCR7 axis may serve as a critical node linking local gut inflammation to the systemic inflammatory load, potentially increasing the risk or severity of insomnia. Consequently, the functional involvement of CCR7 in shared immune dysregulation across insomnia and IBD warrants further mechanistic investigation.

ITGB2 is a gene that encodes the β2 integrin subunit (CD18), which is expressed on leukocytes and mediates immune cell activation and trafficking (46, 47). Dysregulated expression of ITGB2, which critically regulates gut mucosal immunity, may perpetuate chronic intestinal inflammation and disrupt the gut–brain axis, thereby contributing to both IBD pathogenesis and its extraintestinal manifestations (48). Previous studies have shown that ITGB2 expression is significantly upregulated in IBD mouse models and that its suppression alleviates intestinal inflammation, and these results align with our findings. Notably, we have also made a novel observation: ITGB2 is highly expressed in the PBMCs of insomnia patients, a finding further validated in an SD mouse model. Furthermore, we found that ITGB2 expression decreased in resting memory CD4^+^ T cells and increased in activated memory CD4^+^ T cells in both the insomnia and IBD cohorts. ITGB2 is dynamically regulated during T-cell activation, enabling the selective targeting of pathogenic activated memory cells while sparing protective resting cells, making it a promising therapeutic target for patients with IBD and insomnia.

Given the pivotal role of immune dysregulation in the pathogenesis of both insomnia and IBD, we systematically profiled immune infiltration landscapes using three independent deconvolution algorithms (CIBERSORT, EPIC, and xCell), which effectively eliminated the analytical bias inherent to a single-method approach. All three algorithms consistently demonstrated that T cell dysregulation represents a shared immunological feature of IBD and insomnia. γδ T cells emerged as a consistently depleted population across both conditions. CIBERSORT analysis identified decreased γδ T cells in both the IBD and insomnia datasets. This aligns with prior observations that inflamed IBD tissues exhibit significantly lower γδ T cell infiltration compared to non-inflamed tissues (49). Beyond their established roles in intestinal homeostasis, γδ T cells—particularly meningeal populations—have been implicated in neuroimmune modulation of anxiety and, potentially, sleep-related cognition (50), suggesting potential relevance to insomnia pathophysiology, although direct evidence remains limited. CD8^+^ T cells showed a parallel reduction in both diseases by EPIC analysis. In ulcerative colitis, Corridoni et al. demonstrated a marked reduction in tissue-resident memory CD8^+^ T cells (from ~45% to ~10%) alongside expansion of terminally differentiated effector populations, indicating substantial CD8^+^ T-cell remodeling in intestinal inflammation (51). In the context of sleep disruption, chronic sleep deprivation has been shown to reduce CD8^+^ T cell numbers in the tumor microenvironment and impair cytotoxic immunity in an animal model of experimental pulmonary metastasis (52), supporting a broader role for sleep loss in compromising CD8^+^ T cell-mediated immunity. Whether these T cell alterations represent convergent downstream consequences of a shared upstream immune-dysregulation pathway—or reflect distinct tissue-specific responses to chronic inflammation—remains to be determined. Future work should dissect the functional subsets and activation states of these T cell populations in their respective microenvironments, and clarify how peripheral immune alterations translate into tissue-specific pathology. Such efforts may offer new perspectives on the comorbidity of IBD and insomnia and inform the development of immune-modulating strategies targeting shared disease mechanisms. It should be noted that both the immune infiltration estimates and hub gene–immune correlations were inferred from the same bulk transcriptome datasets, rendering them susceptible to cell composition confounding. Although cross-algorithm consistency and cross-disease reproducibility strengthen the robustness of our immunological findings, these computational associations remain to be validated by single-cell or cell-type-specific approaches in independent cohorts.

In conclusion, this study sought to identify hub genes linked to IBD and insomnia while also analysing the possible underlying pathogenesis. Five key genes, IL1B, IRF1, IL7R, CCR7 and ITGB2, are potential biomarkers. However, our study has several limitations. First, this was a microarray data analysis study, and the sample size was relatively limited; larger-scale and multicentre studies are needed for further validation. Second, we conducted experimental verification using mouse models. Although sleep deprivation is a well-established and widely used paradigm to investigate insomnia-related pathophysiology, it cannot fully recapitulate the complexity of clinical insomnia, which may lead to discrepancies between animal results and human conditions. Third, hub genes were validated using separate single-disease models of colitis and sleep deprivation, rather than a unified IBD-insomnia comorbidity model. Given the current lack of standardized and reproducible comorbidity animal models, the independent-model design was adopted to reliably verify the consistent dysregulation of core immune-inflammatory signatures under each pathological condition. Future studies will attempt to establish optimized comorbidity models to further validate the interactive mechanisms between intestinal inflammation and sleep dysfunction. Fourth, although we identified shared systemic inflammatory signatures across blood and intestinal tissues, we cannot reliably discriminate molecular alterations relevant to the interplay of the two disorders from ubiquitous non-specific inflammatory and immune activation. The five hub genes characterized in this work participate in broad immunological processes; these transcriptional changes may simply represent general systemic inflammatory perturbations instead of molecular signals specifically mediating the crosstalk between intestinal inflammation and sleep dysfunction. Further investigations using well-matched clinical cohorts, single-cell transcriptomics, and functional validation are needed to differentiate general inflammation from comorbidity-specific molecular signals. Finally, the drugs predicted in this study have not yet been experimentally verified; relevant investigations will be conducted in future work.

## Conclusion

In summary, this study identified IL1B, IL7R, CCR7, ITGB2 and IRF1 as key shared genes in both IBD and insomnia. Regulatory networks involving these hub genes—namely, TF–miRNA–mRNA interactions—and correlations between hub genes and immune cell infiltration were constructed, and potential candidate drugs were predicted. Our findings provide insights into shared molecular features between insomnia and IBD and offer candidate targets for further experimental and therapeutic exploration.

## Data Availability

The datasets presented in this study are publicly available in the GEO repository. The insomnia dataset (GSE208668) and the IBD dataset (GSE179285) are accessible at https://www.ncbi.nlm.nih.gov/geo/.
