# Supplementary material for: IL1B-centered immune dysregulation involving IL7R, CCR7, ITGB2 and IRF1 across insomnia and inflammatory bowel disease
Source: Front Immunol. 2026 Sep 1;17:1829141. doi: 10.3389/fimmu.2026.1829141 (PMC13575746; doi:10.3389/fimmu.2026.1829141)
Supplement: Supplementary file 1 [file Presentation1.zip › Supplementary Material/Supplementary Figures.pdf]

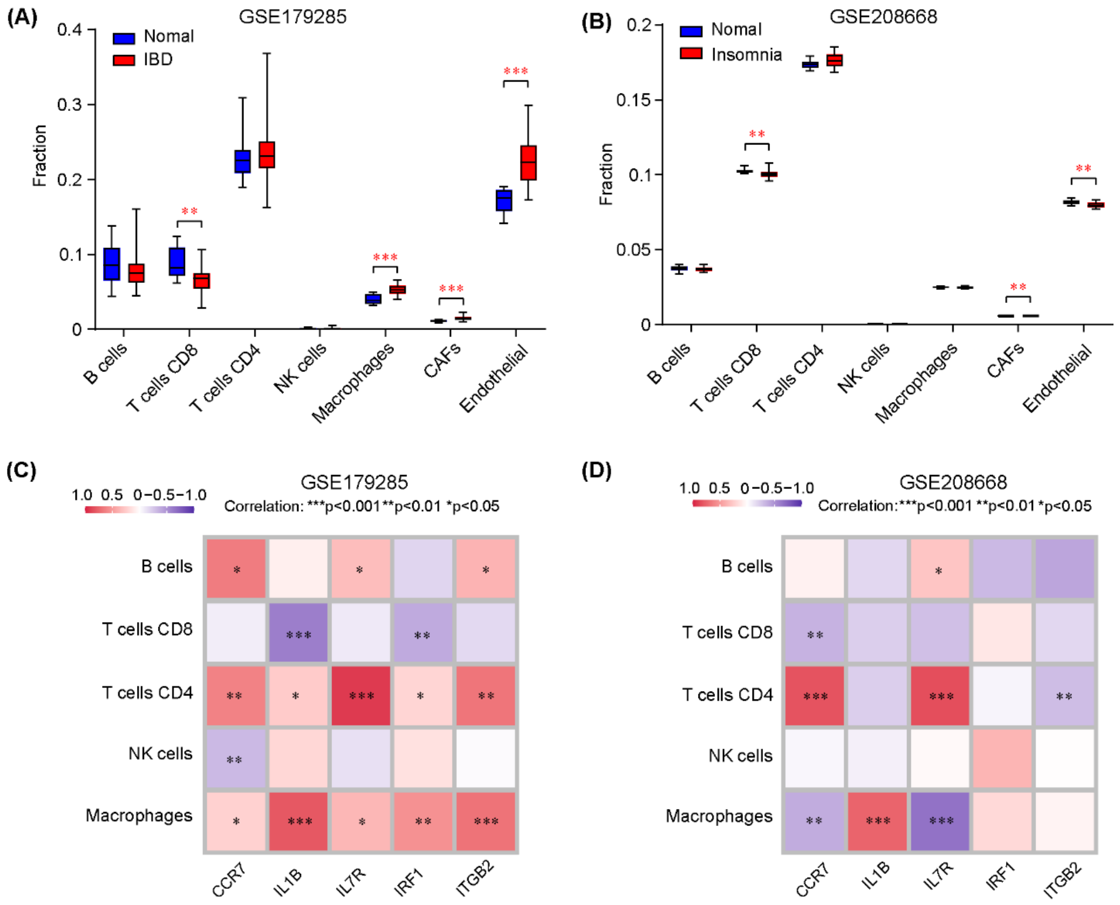

Figure S1 EPIC cellular composition quantification and hub gene–immune correlation analysis in IBD and insomnia cohorts.

(A, B) EPIC-estimated cell fractions in IBD (GSE179285) and insomnia (GSE208668) cohorts. Blue indicates healthy controls, and red indicates patients. Immune cells (B cells, CD8 T cells, CD4 T cells, NK cells, macrophages) and stromal cells (CAFs, endothelial cells) are shown. **(C, D)** Heatmaps showing correlations between hub genes and EPIC-calculated immune cell fractions in the IBD-GSE179285 cohort and the insomnia-GSE208668 cohort. Red indicates positive correlations, and blue indicates negative correlations. *P< 0.05; **P< 0.01; ***P< 0.001.

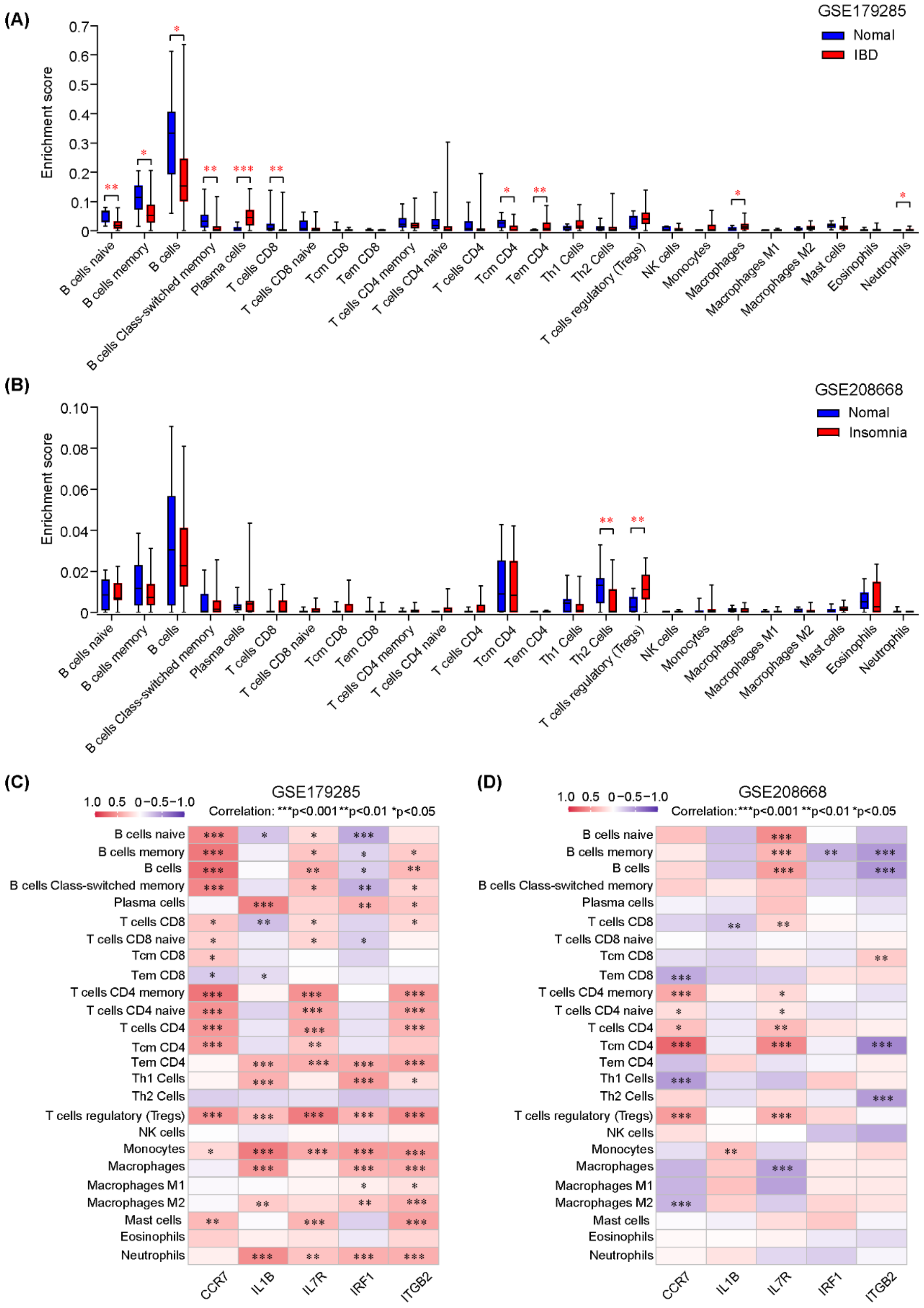

Figure S2 xCell immune infiltration profiling and hub gene–immune correlation analysis in IBD and insomnia cohorts.

(A, B) xCell enrichment scores of immune cells in GSE179285 (IBD) and GSE208668 (insomnia). Blue indicates healthy controls, and red indicates patients. **(C, D)** Heatmaps of correlations between hub genes and xCell-derived immune cell enrichment scores in the IBD-GSE179285 cohort and the insomnia-GSE208668 cohort. Red for positive correlations, blue for negative correlations. *$P <$

0.05; $^{**}P < 0.01$; $^{***}P < 0.001$.
